# Experimental Study on Water-in-Oil Emulsion Stability Induced by Asphaltene Colloids in Heavy Oil

**DOI:** 10.1021/acsomega.4c11723

**Published:** 2025-04-01

**Authors:** Fatemeh Mahmoudi Alemi, Saber Mohammadi

**Affiliations:** † Petroleum Engineering Department, Research Institute of Petroleum Industry (RIPI), Tehran 14856-13111, Iran; ‡ Department of Geosciences, Norwegian University of Science and Technology (NTNU), S. P. Andersens veg 15A, 7031 Trondheim, Norway

## Abstract

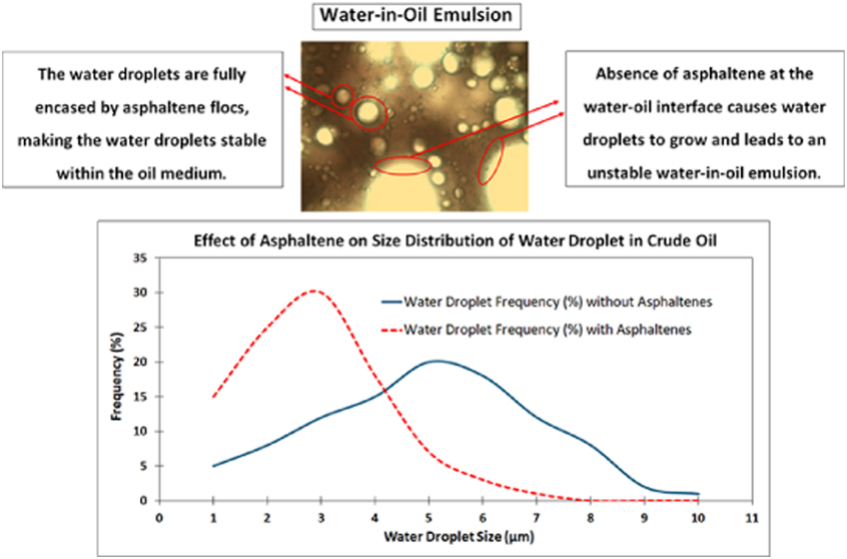

Emulsion stability in water−oil systems is critical for numerous industrial applications, particularly in oil recovery and transportation. However, predicting and controlling the stability of these emulsions under varying conditions remains a challenge. In this study, we investigated the formation and stability of water−oil emulsions under both atmospheric and high-pressure, high-temperature (HPHT) conditions, with a focus on the role of asphaltenes and interfacial tension (IFT). Emulsion stability tests revealed that emulsions exhibit consistent long-term stability at atmospheric conditions, irrespective of the oil−water ratio. Under HPHT conditions, stability varied significantly, with the 75:25 oil−water ratio exhibiting temporary stability, while 50:50 and 25:75 ratios consistently produced unstable emulsions. IFT measurements showed that at atmospheric conditions, crude oil−formation water had an IFT of 21.8 dyn/cm, while crude oil−distilled water had an IFT of 19.8 dyn/cm. Under HPHT conditions (225 °F, 4500 psi), the IFT of live oil−formation water was 18.8 dyn/cm, while live oil−distilled water was 8.0 dyn/cm. At atmospheric conditions, the average IFT was 20.8 dyn/cm, while under HPHT conditions (225 °F, 4500 psi), the average IFT decreased to 13.4 dyn/cm, indicating significant thermodynamic effects on interfacial behavior. Formation water was found to reduce asphaltene precipitation and enhance emulsion stability, emphasizing its role in mitigating asphaltene-related challenges. These findings highlight the importance of tailoring EOR strategies to specific reservoir conditions, including pressure, temperature, oil composition, and water content/chemistry, to optimize emulsion stability, oil displacement, and recovery rates. Future research should explore extended-duration emulsion stability and diverse environmental conditions to better understand their long-term behavior in petroleum applications.

## 1 Introduction

Water-in-oil emulsions are commonly encountered in the production and transportation of heavy crude oils.^1−4^ These emulsions are composed of fine water droplets dispersed throughout a continuous oil phase, stabilized by naturally occurring surfactants like asphaltenes, resins, carboxylic acids, and other polar compounds.^5−9^ The stability of water−oil emulsions is significantly influenced by the presence and interaction of mentioned surface-active components, (e.g., asphaltenes, resins) at the oil−water interface. Asphaltenes, being high molecular weight, aromatic hydrocarbon fractions, are known to adsorb strongly at the interface, forming viscoelastic films that act as physical barriers to coalescence.^10−14^ This ability to form rigid interfacial layers is crucial for stabilizing emulsions, particularly under varying temperature and pressure conditions.^15−21^ Resins, which are structurally similar to asphaltenes but with lower molecular weight, play a complementary role by enhancing the flexibility of these interfacial films and improving their stability.^22,23^ The combined adsorption of asphaltenes and resins reduces interfacial tension (IFT) by forming a cohesive film that prevents the merging of dispersed water droplets.^24−31^ Emulsion stability in heavy oils, especially under high pressure- high temperature (HPHT) conditions, relies on interfacial adsorption and film formation. The complex mixture of polar compounds at the oil−water interface plays a key role, impacting oil recovery, transportation, and refining efficiency. Understanding stability mechanisms is essential for optimizing these processes.

Heavy crude oils, with their high viscosity and complex compositions, pose specific challenges for emulsion stability.^32−34^ The viscosity of heavy oils slows water droplet movement, thereby reducing coalescence and sedimentation rates.^35−37^ Asphaltenes play a central role in stabilizing water-in-oil emulsions through interfacial and colloidal mechanisms, including interfacial adsorption, viscoelastic film formation, colloidal aggregation, and electrostatic repulsion.^14,26,38−47^ The polar regions of asphaltenes interact with water, while the hydrophobic parts remain in the oil phase, reducing interfacial tension and inhibiting droplet coalescence.^5,42,47−50^ At the interface, asphaltenes form rigid, viscoelastic films around water droplets, acting as barriers to maintain separation, with the mechanical strength of these films being essential for preventing droplet merging.^10,24,38,40,41,44,51^ Additionally, asphaltene aggregates in the oil phase adsorb onto water droplets, offering steric stabilization. Their polar functional groups impart a charge that creates electrostatic repulsion, further hindering coalescence.^5,12,52−56^ These aggregates also increase oil phase viscosity, especially in heavy oils, stabilizing emulsions through mechanisms like (1) steric stabilization, where adsorbed aggregates prevent droplet contact, and (2) network formation, where asphaltenes form a structure that traps water droplets, restricting movement.^44,57−61^ A deep understanding of these mechanisms is key to developing effective demulsification strategies and enhancing crude oil recovery and processing.

Current research on emulsion stability is largely limited to specific heavy oil types and controlled lab conditions with dead oil samples, reducing applicability to real-world scenarios.^5,8,62,63^ There is a lack of studies on live oil systems, especially under HPHT conditions, leading to oversimplified experiments that miss critical variables like oil−water ratios, shear rates, and reservoir dynamics. Short experiment durations, weak statistical analyses, and limited insights into brine composition effects on asphaltene aggregation further hinder progress.^64,65^ These gaps undermine effective emulsion management and enhanced oil recovery, highlighting the need for more comprehensive and credible research.

The key goals of this study are to (1) investigate the stability behavior of water-in-oil emulsions induced by asphaltene colloids in both dead and live heavy oil media through comprehensive experimental analysis, (2) evaluate the impacts of critical variables, including oil−water ratio, shear conditions, pressure, temperature, and duration, on emulsion stability, (3) elucidate the underlying mechanisms that stabilize water-in-oil emulsions, (4) address the limitations identified in previous studies by focusing on live oil scenarios to enhance the applicability of the results to real operational conditions, and (5) contribute to improving the efficiency of oil production and processing by advancing the understanding of asphaltene-emulsion interactions.

## 2 Materials and Methods

To investigate the potential for asphaltene deposition and emulsion formation during the water flooding process, a series of experiments were designed based on past experiences. These experiments aimed to measure the equilibrium percentage of solids with liquid and gas phases in the mixture of injection water and reservoir oil, as well as to assess surface tension and emulsion stability. This work requires the study of liquid−gas equilibrium separately based on PVT experiments such as phase separation and the calculation of the physical properties and composition of the oil and gas. How these experiments were conducted is described in the thermodynamics and fluid properties study section. After thermodynamics and fluid property experiments, we will describe experiments related to solid equilibrium, interfacial tension of oil and water, and emulsion stability. The details of the procedure for the main experiments are outlined in the following sections.

### 2.1 Oil and Water Sampling

Crude oil and live oil samples from an oil reservoir are used in this work for the designed experiments. This reservoir suffers severely from water-in-oil emulsion-related issues during the production stage. Therefore, oil samples from this reservoir are well-suited for this study.

One-Phase Sampler (OPS) technology, also known as the Single-phase Reservoir Sampler (SRS), *LEUTERT* model, was employed to collect a representative oil sample. This technique is particularly suitable for asphaltene-related studies as it maintains the fluid pressure at or above the original reservoir level during both transfer and retrieval.^66^ This is achieved through the release of a preset nitrogen charge within the OPS tool, which prevents the sample from undergoing phase separation. Additionally, to ensure temperature consistency during sampling and transfer, heating jackets are utilized, allowing safe sample transfers up to reservoir temperature.^13,67^ This is crucial for preserving the integrity of the oil sample and preventing asphaltene precipitation due to temperature fluctuations. We emphasize that the OPS technology was selected because it maintains the oil sample in a monophasic state, eliminating potential errors related to asphaltene irreversibility. In contrast, other sampling methods like the Positive Displacement Sampler (PDS) are less reliable for studies involving asphaltenes, as they may induce changes in the sample’s phase state, leading to inaccuracies. Crude oil samples, free of associated gas, were collected from a vertical surge tank (VST) located at the wellhead of the reservoir under consideration in this study. The VST, designed for H_2_S service, is a vessel used for storing liquid hydrocarbons postseparation. It also serves to measure liquid flow rates and to determine the combined shrinkage and meter factor.

Here, a formation water sample named as W_F_ is used for the related experiments. A specified volume of formation water was collected based on experimental needs and filtered to eliminate solid-phase impurities. The concentration of different ions (SO_4_^2−^, HCO_3_^−^, Ca^2+^, and Ba^2+^, etc.) was determined using ion titration. Each experiment was repeated three times, and the average of the results was used. The injection water was synthesized based on water flooding plans in an EOR-pilot designed for the studied oil reservoir. The properties and characteristics of the water sample are given in Table 1.

**Table 1:** Characteristics of Formation Water Samples

tests	test method	unit	result
Na^+^	AAS	mg/Lit	63,000
K^+^		mg/Lit	660
Ca^2+^		mg/Lit	8900
Mg^2+^		mg/Lit	2520
Sr		ppm	571
Li		ppm	11
Cl^−^	potentiometry	mg/Lit	116,000
SO_4_^2−^	gravimetry	mg/Lit	510
alkanity as HCO_3_^−^	potentiometry	mg/Lit	232
TDS	gravimetry	ppm	192,404
conductivity@ambient Temp	ASTM D1125	ms/cm	198
pH@amb.Temp	ASTM E70	-----	7.01
SP.Gr@20 °C/20 °C	ASTM D4052	-----	1.1219

### 2.2 Characteristics of the Oil Sample

PVT experiments on the oil sample are executed to determine how the oil behaves under different pressure and temperature conditions, and they provide crucial data for reservoir engineering and production optimization. PVT experiments are essential for understanding reservoir characteristics, estimating reserves, and designing production strategies in the oil and gas industry. In this work, through the PVT study, bubble point pressure, reservoir oil composition, density, differential vaporization data, and viscosity of reservoir oil versus pressure and temperature were obtained. Table 2 shows the main characteristics of the studied oil sample and the results of saturates-aromatics-resins-asphaltenes (SARA) analysis. SARA analysis has been performed based on ASTM D2007−91,^68^ which is based on clay-gel adsorption chromatography and thin-layer chromatography with flame-ionization detection (TLC-FID). Typically, prior to conducting costly and time-intensive HPHT experiments for asphaltene, reservoir fluids are screened to assess the likelihood of asphaltene precipitation. The most commonly used screening criteria include the colloidal instability index (CII) and the De Boer plot.^69,70^ The CII is defined as the ratio of the sum of asphaltenes and saturates fractions of crude oil to the sum of aromatics and resins.^69^ The De Boer method utilizes a graphical plot based on solubility concepts to predict the thermodynamic conditions under which asphaltene precipitation/deposition occurs.^71^ In the De Boer method, the difference between reservoir pressure and bubble point pressure is plotted against reservoir fluid density.^72^ For the oil sample studied here, the CII value (reported in Table 2) is approximately 1.06, confirming the instability of the oil sample regarding asphaltene precipitation. According to the De Boer method, the oil sample falls within unstable regions, indicating it is categorized as unstable/problematic oil prone to solid phase issues during reservoir production.

**Table 2:** Properties and Specifications of the Oil Sample

component	unit	value
H_2_S	mol %	0.08
N_2_	mol %	1.18
CO_2_	mol %	0.80
C_1_	mol %	25.25
C_2_	mol %	8.60
C_3_	mol %	6.46
iC_4_	mol %	1.47
nC_4_	mol %	4.81
iC_5_	mol %	1.94
nC_5_	mol %	2.18
C_6_	mol %	4.94
C_7_	mol %	2.90
C_8_	mol %	2.75
C_9_	mol %	3.16
C_10_	mol %	3.23
C_11_	mol %	3.30
C_12+_	mol %	26.95
molecular weight of residual oil	g/mol	303
molecular weight of C_12+_ fraction	g/mol	474
molecular weight of reservoir oil	g/mol	168
solution gas oil ratio	SCF/STB	371.8
reservoir temperature (*T*_R_)	°F	225
reservoir pressure (*P*_R_)	psia	4830
bubble point pressure (*P*_b_)	psia	1613
gravity of dead oil	API	16.2
saturates	mass%	46.5
aromatics	mass%	33.2
resins	mass%	15.3
asphaltenes	mass%	5.0
colloidal instability index (CII)	-	1.06

### 2.3 HPHT Filtration Experiment

**Figure 1:**
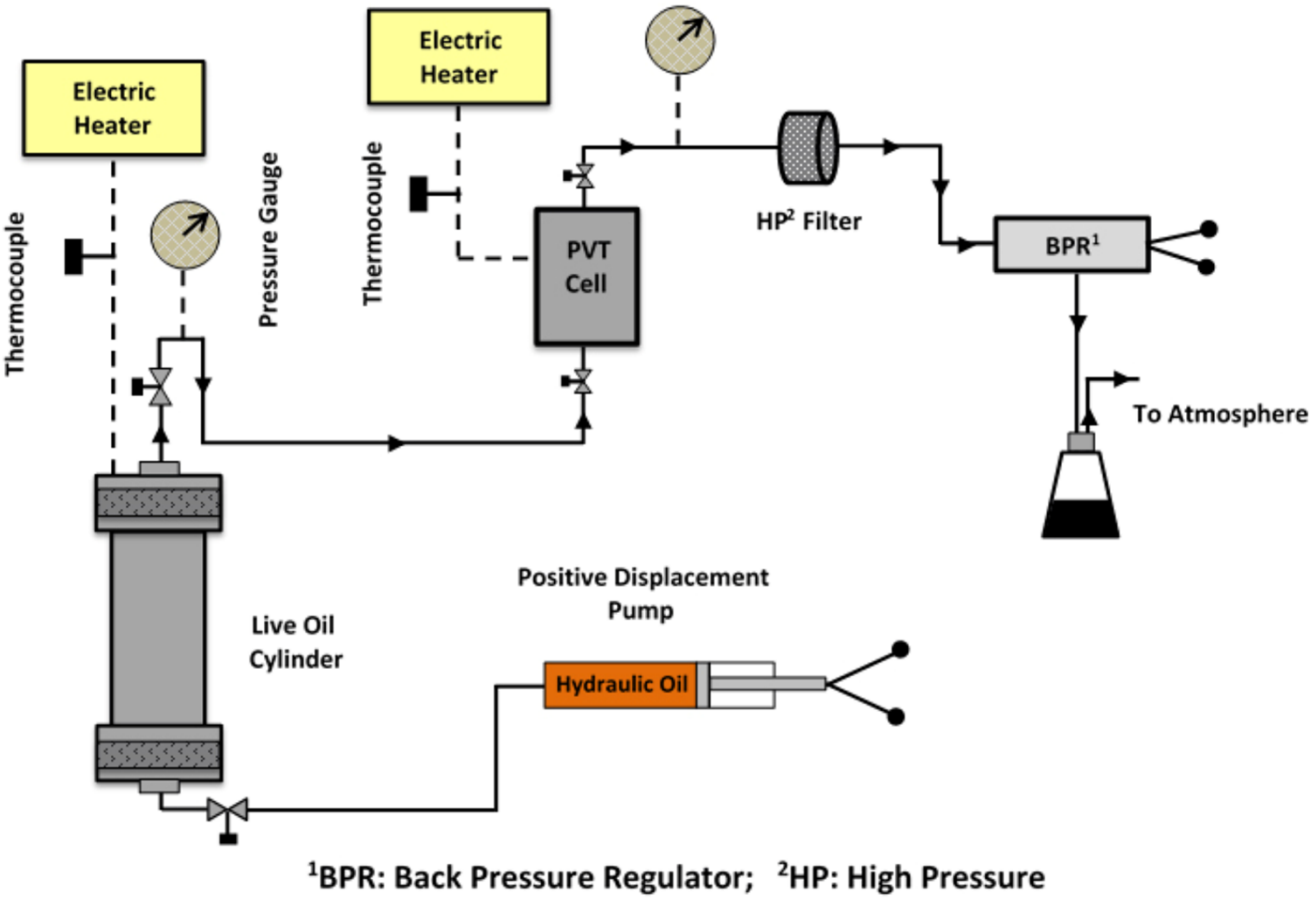
Schematic diagram of the HPHT filtration setup used for asphaltene precipitation study.

The HPHT filtration experiments are performed to measure the amount of precipitated asphaltene in live oil versus pressure at constant temperature. By filtration experiments, the stability or dispersion of asphaltene aggregates in the bulk of the oil phase can be evaluated. HPHT filtration tests are conducted with a 0.20 μm Cellulose Nitrate filter. The schematic diagram of the HPHT filtration setup is depicted in Figure 1. To initiate the test, approximately 240 cm^3^ of single-phase reservoir fluid is transferred into the high-pressure PVT cell under constant pressure and temperature. Each experiment begins with the PVT cell being stabilized at the test’s initial pressure and temperature for about 4 days. Following stabilization, the pressure is incrementally reduced in predefined steps above and below the saturation pressure. At each step, the cell’s contents are homogenized for 24 h. Subsequently, the fluid is filtered, and a small, well-mixed sample (20 cm^3^) is expelled from the PVT cell under experimental conditions to flash to atmospheric pressure. The asphaltene content is then measured using the IP-143 standard test (ASTM D6560−00)^73^ to determine the precipitated asphaltene amount. The HPHT filtration setup can operate at pressures up to 12,000 psi and temperatures up to 350 °F. Detailed description of the experimental procedure can be found in published articles by the authors.^74^

### 2.4 Emulsion Formation and Stability at Ambient and HPHT Conditions

For investigation of emulsion formation and stability at ambient conditions, the crude oil and water under study are mixed using a mechanical stirrer at a fixed speed. After a specified time, the stirring process is stopped, and the tendency of the phases to form an emulsion or to separate is visually observed. The stirrer used in this work is an *IKA-WERKE* model *EURO-ST P CV P1*, capable of rotating from 50 to 1200 rpm, equipped with a 5 cm diameter blade. First, the crude oil sample is shaken well to homogenize it. Then, 50 mL of the sample are poured into a 200 mL beaker with an 8 cm diameter, and 50 mL of water is added. The sample is stirred with the mechanical stirrer at the desired speed (250, 500, 750, or 1000 rpm) using a 5 cm diameter blade for 15 min. Finally, the formed emulsion is transferred to a 100 mL test bottle to assess its stability. After 24 h, samples are taken from the top of the test bottle for microscopic imaging.

**Figure 2:**
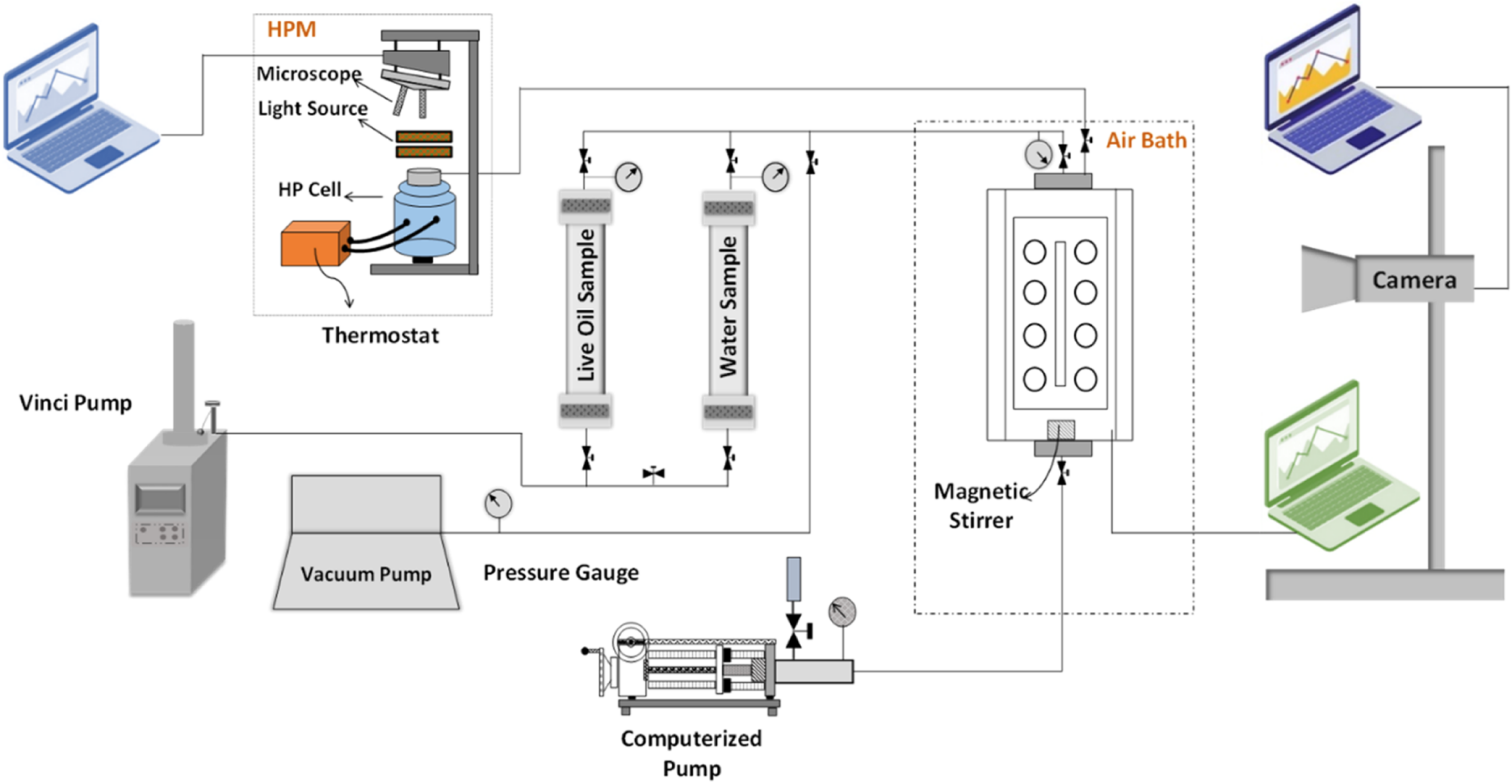
Schematic diagram of the HPHT assembly for studying the formation and stability of water-in-oil emulsions under reservoir conditions.

Producing finely dispersed emulsions under HPHT conditions requires specialized facilities. Our research group designed and fabricated an HPHT emulsion assembly comprising several components: a main visual cell, HPHT cylinders, a mixer, various water and oil inlet valves, a high-pressure heater and thermometer, a pressure gauge, a high pressure microscope (HPM), pumps, a PC, and more. Figure 2 illustrates the schematic diagram of the HPHT setup used for forming stable water-in-oil emulsions. As shown in the figure, two separate cylinders with distinct lines were used for storing live oil and formation water.

After calculating various water-in-oil mixtures, water is injected first, followed by oil, into the main cell. The fluids are then thoroughly mixed using a magnetic stirrer at a specific rotational speed. Before mixing the fluids, both the oil and water in the cylinders are maintained at a constant temperature of 225 °F and an initial pressure of 4500 psi. The test conditions were selected based on the initial pressure and temperature of the oil reservoir, as well as the saturation pressure of the reservoir fluid. It is crucial to ensure that the rotation of the main cell is configured to prevent the magnetic forces from directly influencing the oil and to avoid the deposition of solids, such as asphaltene particles, in the live oil medium. The two fluids must be completely mixed in the main cell before increasing the pressure and temperature to the desired values. Subsequently, the cell is stirred for about 24 h at the desired pressure and temperature to achieve a homogeneous emulsion. During the mixing of the two phases, the inlet valve above the main cell is kept closed to prevent any pressure drop in the system. The equilibrated fluid inside the main cell is then monitored with a camera to verify the formation of a stable water-in-oil emulsion. To analyze the distribution of water droplets in the emulsion, a subsample of the homogenized emulsion is transferred from the main cell into the HPM cell. Using the HPM, the growth of asphaltene particles and water-in-oil droplets, as well as their morphology during pressure decline, can be monitored. The HPM consists of a HPHT cell featuring two sapphire windows, a light source, and a precise microscope (Leica Z16 APO, Germany) positioned above the cell. The cell has a thickness of 0.3 mm and a sapphire diameter of 9.2 mm. Temperature control is achieved with a circulating silicone oil bath from HUBER-GmbH, Germany. A positive displacement pump is employed to maintain the pressure within the HPHT cell. Throughout the experiment, the contents of the HPM cell are observed, and micrographs are captured using a video camera. High-resolution images of the fluid sample inside the cell are recorded at specific pressure intervals to track changes in emulsion bubble sizes and their interactions with asphaltene flocs. The Java-based *ImageJ* software package (version 1.6), developed at the National Institutes of Health, is utilized to process the obtained micrographs.^75^ Images from the HPM cell are analyzed to determine the size distribution of the water-in-oil droplets and to monitor the water−oil interfaces within the live oil bulk medium. The repeatability error in the mean droplet area calculation is ±2 μm^2^. The HPM assembly operates at a maximum pressure of 15,000 psi and a temperature of 340 °F. Details of the HPM procedure can be found elsewhere.^76^ During the image analysis, outlier data were excluded to enhance accuracy. If different droplet distributions were observed at various locations, the analysis was repeated using images from multiple points within the sample.

### 2.5 Interfacial Tension Measurements

The dynamic IFT at the water−oil interface containing asphaltenes was measured using the pendant drop technique, employing the *IFT* 7*00-HPHT* Interfacial Tension Meter, manufactured by *Vinci*, France. This device is specifically designed to measure the IFT between two immiscible fluids under simulated reservoir conditions using either the pendant or rising drop methods. Additionally, it can determine the contact angle of a liquid droplet on a solid surface via the sessile drop method. The system operates under conditions that replicate reservoir pressure and temperature, providing a realistic environment for testing. In the pendant drop method, a droplet of one fluid (i.e., water) is formed at the tip of a capillary needle within a chamber filled with another fluid (i.e., oil). The droplet is subjected to the desired pressure and temperature conditions. Advanced image capture and processing systems are then used to record the shape of the droplet and compute geometric parameters needed to calculate the interfacial tension based on the *Laplace* equation. When equilibrium is achieved, the contact angle is directly measured using *Vinci* interpretation software, ensuring precise measurements. The pendant drop technique was specifically selected for this study because of its high precision and its capability to simulate HPHT conditions encountered in oil reservoirs. This method allows for dynamic IFT measurements, which are critical for understanding the behavior of live oil and water interactions under realistic reservoir conditions. By replicating these conditions, the pendant drop method ensures that the IFT data obtained is accurate and relevant, providing essential insights into the stability of water-in-oil emulsions and the role of asphaltenes in stabilizing these emulsions. Such precision is crucial for assessing the efficiency of EOR processes and predicting potential challenges during water flooding operations.

In this study, it is noted that the pendant drop method measures IFT by immersing a water droplet in a bulk oil phase that includes emulsified droplets at different oil−water ratios (75:25, 50:50, and 25:75). The presence of dispersed water droplets in the bulk oil affects the measured IFT, as higher water content increases the interfacial area requiring stabilization by asphaltenes and other surface-active compounds. Consequently, at higher water ratios, the measured IFT reflects the distribution of asphaltenes across both the bulk oil and the primary oil−water interface, influencing overall emulsion stability. For the live oil IFT measurements, an equilibrium time of 24 h was selected. This duration was determined based on preliminary observations and existing literature, which indicate that 24 h is sufficient to achieve stable readings, similar to the dead oil measurements.

### 2.6 Selection of Oil−Water Ratios

The oil−water ratios of 75:25, 50:50, and 25:75 were selected to systematically examine the impact of varying water contents on emulsion stability under HPHT conditions. These ratios simulate a range of operational scenarios encountered in oil recovery, where water cut (the proportion of water in the produced fluid) can vary significantly. By assessing these ratios, we aim to understand how different water levels affect the formation and stability of water-in-oil emulsions in an environment that mimics actual reservoir conditions.

- Oil−Water Ratio of 75:25: This dominant oil phase ratio is common in the early production stages or reservoirs with minimal water intrusion. Studying this ratio helps us understand how asphaltenes, acting as natural stabilizers, perform when the oil phase is significantly larger. This insight is crucial for determining the need for additional stabilizing agents.
- Oil−Water Ratio of 50:50: This balanced ratio allows us to evaluate when water content begins to significantly influence emulsion behavior, particularly in midproduction phases or during water flooding. It helps assess the interplay between oil and water and the effectiveness of asphaltenes in stabilizing droplets under equal phase conditions.
- Oil−Water Ratio of 25:75: This ratio represents high water cut scenarios commonly seen in mature fields or during aggressive EOR water flooding. It allows us to investigate the limits of asphaltene interaction at the interface and its effect on droplet coalescence and emulsion stability. It also highlights the potential challenges in oil recovery, such as emulsion destabilization and increased processing costs.

These ratios allow us to capture a comprehensive range of conditions, enhancing our understanding of water-in-oil emulsion stability mechanisms influenced by asphaltenes and water levels. This knowledge is essential for optimizing EOR strategies and addressing operational challenges during water flooding and production. Additionally, these findings may identify thresholds for necessary interventions, such as chemical stabilizers or adjustments in water flooding rates, to maintain long-term emulsion stability and prevent production inefficiencies.

## 3 Results and Discussion

### 3.1 Formation of Emulsion at Atmospheric Conditions

To investigate the potential for emulsion formation from the mixing of oil and water, repeated emulsion stability tests were conducted using a mechanical stirrer. These tests were initially performed to determine the minimum speed range required for emulsion stability and subsequently to examine the effect of mixing percentage (i.e., oil−water ratios) on stability. The results of these tests are presented in Table 3. The results of the various emulsion stability tests under atmospheric conditions using a mechanical stirrer reveal consistent stability across different oil−water ratios and over various time intervals. Across all three oil−water ratios, the emulsions demonstrated stability at 1000 rpm. This suggests that the emulsions formed were robust and resilient to phase separation under the given experimental conditions. In addition to this work, a stirring speed of 1000 rpm was selected based on preliminary tests and prior studies, which showed it to be optimal for ensuring consistent mixing without introducing excessive shear that could potentially alter the interfacial properties under the experimental conditions. The oil−water ratio does not seem to significantly impact the stability within the tested range. All ratios showed equivalent stability, indicating that, at least for the range of ratios tested, the proportion of oil to water does not critically affect emulsion stability. The stability observed from 5 min to 2 weeks demonstrates that the emulsions are not only temporarily stable but also exhibit long-term stability under atmospheric conditions with mechanical stirring. This long-term stability is crucial for practical applications where emulsions need to remain stable over extended periods.

**Table 3:** Results of Various Emulsion Stability Tests under Atmospheric Conditions Using a Mechanical Stirrer

emulsion components	oil−water ratio, MR (vol %)	angular velocity (rpm)	5 min	10 min	30 min	2 h	24 h	2 weeks
oil−formation water	50:50	1000	stable	stable	stable	stable	stable	stable
oil−formation water	75:25	1000	stable	stable	stable	stable	stable	stable
oil−formation water	25:75	1000	stable	stable	stable	stable	stable	stable

The findings are beneficial for industries involved in oil recovery and transportation, where stable emulsions of oil and water are often required to ensure efficient and predictable handling and processing. While water−oil emulsions can contribute to oil mobilization under certain conditions, we acknowledge that this effect may vary in real reservoir environments. Emulsion stability can help block high-permeability channels, encouraging a more uniform sweep of oil through lower-permeability zones, which can improve oil recovery. However, in practice, the formation and behavior of water−oil emulsions are influenced by various factors, including oil composition, reservoir conditions, and operational parameters. The consistent stability across varying oil−water ratios and time frames supports the reliability of using mechanical stirring at 1000 rpm to maintain emulsion stability in various chemical engineering processes. While the presented data is comprehensive, with measurement up to 2 weeks, it would be advantageous to investigate the stability over longer periods to fully understand the long-term behavior of these emulsions. Future studies could explore different angular velocities and other environmental conditions (such as temperature and pressure variations) to optimize and expand the application range of these stable emulsions. In summary, the tests show that under atmospheric conditions with a mechanical stirrer operating at 1000 rpm, emulsions with oil−formation water in different ratios remain stable for at least 2 weeks, suggesting good potential for various industrial applications where such stability is required. While our study focused on a fixed stirring speed range, evaluating lower stirring speeds could provide a better understanding of the minimum energy required for emulsion stabilization. Typically, at lower stirring speeds (<500 rpm), emulsions may not form or may exhibit lower stability due to insufficient shear forces required to disperse water droplets uniformly within the oil phase. Future work will explore these thresholds systematically to optimize energy-efficient stirring conditions for field applications.

### 3.2 Emulsion Formation under HPHT Conditions with Live Oil

To investigate emulsion stability/formation phenomenon under reservoir pressure and temperature conditions with the live oil sample, this test was conducted in a pressurized cell (Figure 2). The obtained results are given in Table 4.

**Table 4:** Results of Various Emulsion Stability Tests of Formation Water and Live Oil at a Temperature of 225 °F and Pressure of 4500 psi

emulsion components	oil−water ratio (vol %)	angular velocity (rpm)	5 min	10 min	30 min	2 h	24 h
oil−formation water	50:50	1000	unstable	unstable	unstable	unstable	unstable
oil−formation water	75:25	1000	stable	stable	stable	stable	unstable
oil−formation water	25:75	1000	unstable	unstable	unstable	unstable	unstable

The results of the emulsion stability tests of formation water and live oil at a temperature of 225 °F and a pressure of 4500 psi show significant variations in stability depending on the oil−water ratio. For 50:50 oil−water ratio, the emulsion was unstable at all observed time intervals (5 min to 24 h). This indicates that an equal proportion of oil and formation water does not favor stable emulsion formation under the given conditions. For 75:25 oil−water ratio, the emulsion was stable up to 2 h but became unstable by 24 h, which suggests that a higher proportion of oil contributes to initial emulsion stability, but the stability degrades over a longer period. For 25:75 oil−water ratio, the emulsion was unstable at all observed time intervals (5 min to 24 h). A higher proportion of formation water also does not favor stable emulsion formation under the given conditions. The instability of the emulsion with a higher formation water fraction can be attributed to the reduced availability of asphaltenes to sufficiently stabilize the larger volume of water droplets.^14,44^ As the water content increases, there is a greater surface area at the oil−water interface, which requires more asphaltenes to form the stabilizing films. When the concentration of asphaltenes is insufficient to cover the increased surface area, the films become weaker or incomplete, allowing water droplets to coalesce, leading to emulsion instability. Additionally, the higher water fraction can increase the likelihood of phase separation due to density differences between oil and water.

Both ratios (50:50 and 25:75) resulted in unstable emulsions across all time intervals, indicating that neither an equal proportion nor a higher water content is conducive to emulsion stability in this scenario. In a 75:25 ratio, the initial stability indicates that a higher oil content can promote the formation of stable emulsions; however, this stability is only temporary. Over time, the emulsion tends to break down, likely due to the coalescence of droplets or separation due to the oil−water density differences.

High temperature (225 °F) can reduce the viscosity of the oil, making it more likely to coalesce and separate from water over time. High pressure (4500 psi) can impact the solubility of gases in the oil and water, potentially affecting interfacial tension and stability. Stability at shorter time intervals (up to 2 h) versus instability at 24 h highlights the importance of considering the duration for which emulsion stability is required in practical applications. For applications where emulsions need to remain stable for short periods, a higher oil content (75:25) could be suitable. However, for long-term stability, additional measures (e.g., stabilizing agents) might be necessary. Understanding the stability behavior helps in designing processes that require emulsions to either remain stable or break down at specific stages. The stability of emulsions formed from formation water and live oil under HPHT conditions varies significantly with the oil−water ratio. While a higher oil content (75:25) can initially stabilize the emulsion, this stability does not persist over 24 h. Equal (50:50) and higher water content (25:75) ratios consistently produce unstable emulsions.

### 3.3 Interfacial Tension (IFT) of Emulsions at Atmospheric and HPHT Conditions

The IFT results between crude/live oil and formation/distilled water under various pressure and temperature conditions are crucial for understanding fluid interactions in petroleum reservoirs, particularly with asphaltene-containing fluids. Distilled water is used as a controlled baseline to isolate the live oil’s behavior at the oil−water interface, enabling a clearer analysis of the impact of specific ions in formation water on emulsion stability and IFT. The results of IFT measurement for different fluids and test conditions are given in Table 5.

**Table 5:** Results of Interfacial Tension (IFT) between Crude Oil/Live Oil and Formation Water (50:50) under Atmospheric and HPHT Conditions^a^^,^^b^

pressure (psi)	temperature (°F)	oil type	IFT_Oil-FW_* (dyn/cm)	IFT_Oil-DW_* (dyn/cm)
14.7	75	crude oil	21.8	19.8
1700	225	live oil	17.7	8.6
3000	225	live oil	18.5	8.1
4500	225	live oil	18.8	8.0

a IFT_Oil-FW_*: IFT for oil−formation water.

b IFT_Oil-DW_*: IFT for oil−distilled water.

At atmospheric pressure and room temperature, IFT is higher for both oil−formation water and oil−distilled water systems, with crude oil showing a higher IFT with formation water than distilled water. This is due to stronger intermolecular forces between crude oil and formation water components, driven by interactions between ions (Ca^2+^, Mg^2+^, Na^+^, Cl^−^) and polar components in the oil, like asphaltenes and resins. These ions contribute to a stable, rigid interface by forming structured water layers or ion-pair complexes. In contrast, distilled water, lacking such ions, shows weaker interactions, allowing water molecules to disperse more easily into the oil phase, resulting in lower IFT. Increasing pressure from 1700 to 4500 psi at a high temperature slightly raises IFT with formation water, suggesting increased oil−water cohesiveness. However, IFT with distilled water decreases under pressure, possibly due to reduced gas content in the oil, enhancing oil−water interaction. Crude oil generally exhibits higher IFT with both water types, indicating stronger internal cohesion. Live oil under high-pressure, high-temperature (HPHT) conditions shows lower IFT than crude oil at atmospheric conditions, a beneficial factor for oil recovery as lower IFT improves oil displacement and recovery efficiency.

The slight increase in IFT between live oil and formation water with increasing pressure could impact EOR techniques like water flooding, where reducing IFT is crucial for efficient oil mobilization. Conversely, the decreasing trend of IFT between live oil and distilled water with increasing pressure suggests that under reservoir conditions, changes in the oil−water interaction may influence wettability and phase behavior. The high temperature of 225 °F in HPHT tests mirrors realistic reservoir conditions, with significant IFT reduction, particularly with distilled water, highlighting temperature’s critical role in enhancing oil recovery. Additionally, lowering the salinity of injection water decreases IFT, promoting emulsion formation and improving sweep efficiency during water flooding, making low-salinity water flooding a promising EOR strategy. The contrast in IFT values between crude oil at surface conditions and live oil at HPHT conditions underscores the need to consider reservoir-specific conditions for accurate EOR method optimization. Overall, these findings demonstrate the importance of tailoring EOR strategies to specific reservoir conditions to achieve maximum oil recovery efficiency.

**Figure 3:**
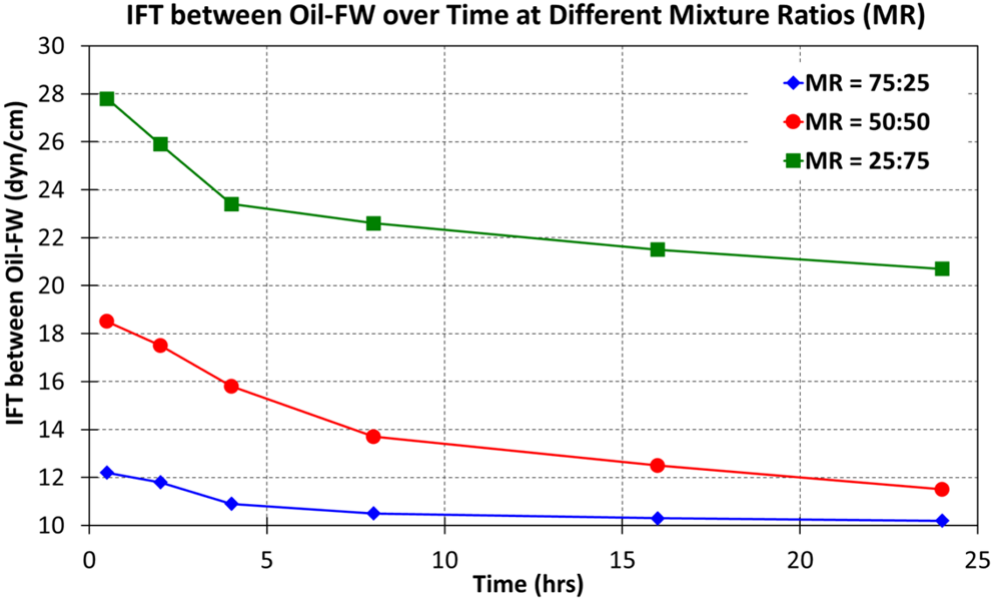
IFT between crude oil and formation water vs time at different oil−water mixing ratios (MR) at constant pressure and temperature (3000 psi, 225 °F).

Figure 3 demonstrates that IFT between oil and formation water decreases over time across different oil−water mixing ratios (MR) at constant pressure and temperature (3000 psi, 225 °F). The MR = 75:25 mixture consistently shows the lowest IFT, suggesting the most stable emulsion, followed by 50:50 and 25:75 ratios. Lower IFT indicates enhanced dispersion and stabilization of water droplets within the oil phase, with higher oil content promoting emulsion stability by effectively encapsulating water droplets. The prolonged lack of stabilization in IFT curves even after 24 h is due to the continuous reorganization of asphaltenes and resins at the oil−water interface. Asphaltenes’ time-dependent adsorption behavior results in viscoelastic films that evolve under the influence of pressure, temperature, and ionic interactions. The complexity of multicomponent interactions under reservoir conditions further extends the stabilization process. The results obtained and the mechanisms discussed here are corroborated by existing research from other scholars.^14,38,44,56^

The IFT values obtained for crude oil in both formation water and distilled water were lower than the commonly reported range for oil−water systems (30−70 dyn/cm). The lower-than-expected IFT values in this study can be explained by a combination of oil composition, water chemistry, and thermodynamic conditions. The high asphaltene and resin content acts as natural surfactants, reducing IFT by forming rigid interfacial films. Additionally, multivalent ions (Ca^2+^, Mg^2+^, SO_4_^2−^, HCO_3_^−^) in formation water interact with polar oil components, further lowering IFT. Under HPHT conditions, dissolved gases alter molecular interactions, while elevated temperatures (225 °F) increase molecular mobility and weaken intermolecular forces, leading to reduced IFT. The use of the pendant drop method, which measures dynamic IFT, may also contribute to lower values compared to equilibrium IFT. These findings emphasize the role of oil−water interactions and reservoir conditions in interfacial behavior, which is crucial for EOR strategies and flow assurance.

### 3.4 Impact of Asphaltene Precipitation on Emulsion Stability in Live Oil under HPHT Conditions

The asphaltene precipitation behavior under HPHT conditions was analyzed using filtration tests at various pressures while maintaining a constant reservoir temperature of 225 °F. The live oil showed a CII of 1.06, suggesting that the oil sample is unstable and has a high risk of asphaltene precipitation and deposition. The study evaluated asphaltene precipitation by measuring weight percentages at four different pressure levels and in two conditions: (i) blank live oil and (ii) live oil with a 50:50 mixture of formation water.

**Figure 4:**
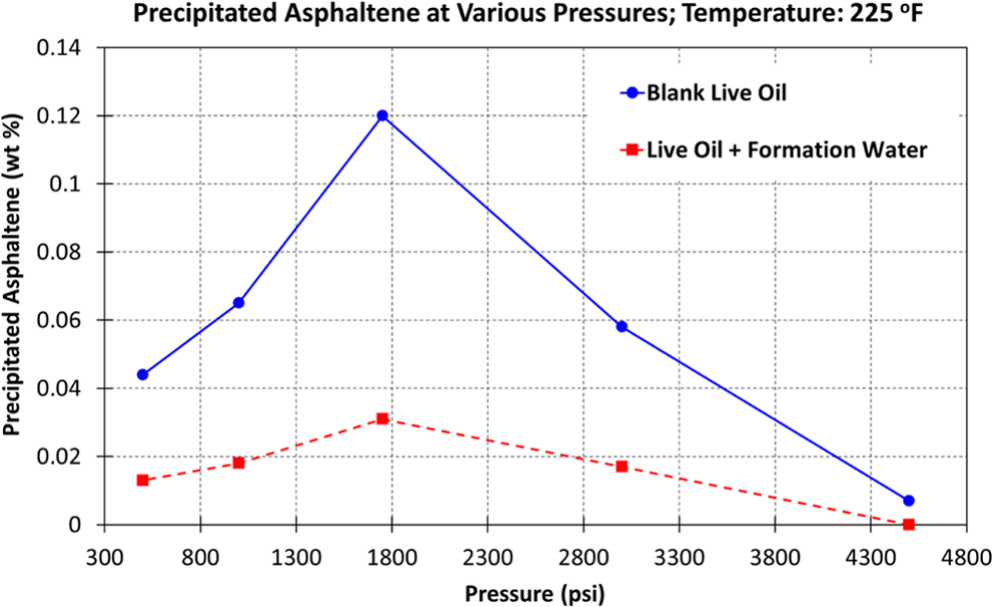
Amount of precipitated asphaltene vs pressure for blank live oil and live oil with formation water as emulsion at constant temperature of 225 °F. This plot illustrates the weight percentage of precipitated asphaltene at varying pressures, highlighting the effect of pressure on asphaltene precipitation. The graphical representation reinforces the conclusion that higher pressures inhibit asphaltene precipitation, while the presence of formation water significantly decreases the amount of precipitated asphaltene.

The results of the weight percentage of asphaltene precipitated in these four scenarios are presented in Table 6. Also, for better comparison, the results are depicted in Figure 4. The results revealed that as the pressure decreases from 4500 to 1750 psi, above the saturation pressure, the amount of precipitated asphaltenes increased significantly in both the blank live oil and the live oil−formation water mixture. This pattern shifted at pressures below the saturation point, where the amount of precipitated asphaltene decreased as pressure was further lowered to 500 psi. At pressures above the saturation point, the oil retains a significant amount of dissolved gases, enhancing asphaltene solubility. As pressure reduces toward and below the saturation pressure, gas liberation and oil expansion promote asphaltene aggregation and precipitation. However, further pressure reduction below the saturation pressure leads to a decrease in precipitation due to reduced oil viscosity and increased phase separation efficiency, which removes the asphaltenes from the bulk phase. The results indicate that the maximum asphaltene precipitation occurs at 1750 psi and 225 °F, which coincides with the bubble point pressure of the system. This trend aligns with prior studies indicating that asphaltene precipitation is highly dependent on phase behavior and pressure depletion. However, it is important to recognize that asphaltene precipitation is not solely governed by the bubble point but also by the Lower Asphaltene Onset Pressure (LAOP) and Upper Asphaltene Onset Pressure (UAOP). These onset pressures define the critical pressure range within which asphaltene precipitation is most likely to occur. Several studies have demonstrated that asphaltene precipitation and deposition exhibit nonlinear behavior under HPHT conditions, particularly in CO_2_-EOR systems.^77,78^ These studies highlight that above the UAOP, asphaltenes remain dissolved, while below the LAOP, the precipitated asphaltenes tend to redissolve due to changes in solubility and molecular interactions. The existence of an optimal pressure window between LAOP and UAOP is crucial for the design of EOR strategies and flow assurance modeling to mitigate asphaltene-related challenges in oil production. To enhance predictive capabilities, future research should incorporate LAOP and UAOP thresholds in asphaltene modeling to refine precipitation behavior under varying reservoir conditions. This will allow for the development of more robust models for asphaltene management and improved mitigation strategies in EOR applications.

**Table 6:** Results of HPHT Filtration Experiments

		blank live oil	live oil + formation water
pressure (psi)	temperature (°F)	precipitated asphaltene (wt %)	precipitated asphaltene (wt %)
4500	225	0.007	0.000
3000	225	0.058	0.017
1750	225	0.120	0.031
1000	225	0.065	0.018
500	225	0.044	0.013

The addition of formation water consistently led to lower asphaltene precipitation across all pressure ranges. This suggests that formation water can controls the asphaltene phase behavior, possibly through interactions that reduce asphaltene aggregation. The role of key factors and the main findings in this section are as follows:

- Pressure Influence: Higher pressures inhibit asphaltene precipitation, with minimal precipitation at 4500 psi. As pressure decreases, reduced asphaltene solubility leads to greater asphaltene precipitation, which is critical for understanding reservoir management and potential challenges during pressure depletion.
- Role of Formation Water: Formation water reduces asphaltene precipitation by stabilizing the oil−water interface. This effect is crucial for enhanced oil recovery techniques involving water flooding, as it indicates that formation water can help control asphaltene behavior and minimize deposition in the reservoir and production equipment.
- Implications for Production: The findings underscore the need to monitor and control pressure levels during oil production to manage asphaltene-related issues effectively. The findings indicate that water−oil emulsions that include formation water can assist in managing asphaltene stability, thereby minimizing the risks of deposition in both the reservoir and production equipment.

### 3.5 Optical Microscopy Analysis

**Figure 5:**
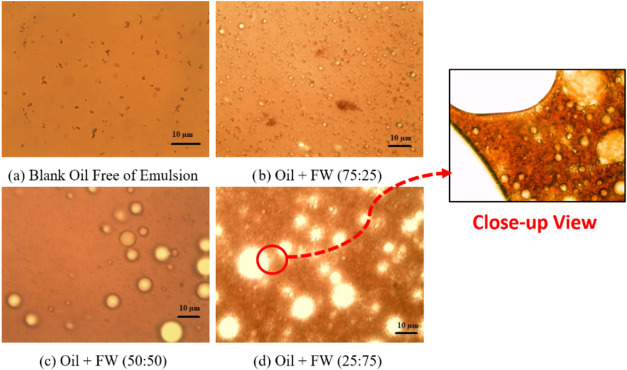
Microscopic images of: (a) blank oil free of emulsion, (b) oil−water emulsion (MR = 75:25), (c) oil−water emulsion (MR = 50:50), (d) oil−water emulsion (MR = 25:75); crude oil was titrated with *nC*_*7*_ as asphaltene precipitant; in picture (d), the oil seems to be highly saturated with small water droplets, indicating a significant emulsion formation.

Figure 5 illustrates various water−oil emulsions with different mixing ratios and the role of asphaltenes in stabilizing these emulsions. Image (a) shows crude oil without any water−oil emulsion, indicating a single-phase system where there is no significant dispersion of water. The absence of emulsion suggests that the crude oil is stable in its pure form due to the lack of emulsifiers or stabilizers. Image (b) depicts an emulsion with a MR of 75:25, where water droplets are dispersed within the oil phase. At this ratio, the stability of the emulsion is influenced by asphaltenes, which can adsorb at the oil−water interface, reducing interfacial tension and forming a film that prevents coalescence of the water droplets. However, a higher oil content, results in smaller water droplets in size. Image (c) depicts an emulsion with a MR of 50:50, featuring a higher number of water droplets and larger droplet sizes, indicating reduced emulsion stability. The MR of 50:50 provides a balanced interaction between oil and water, allowing asphaltenes to effectively stabilize the emulsion by covering more interfaces, thereby preventing coalescence and ensuring a more homogeneous mixture. Image (d) shows an emulsion with a MR of 25:75, where the increased water content leads to the formation of larger water droplets within the oil medium. Consequently, the higher water content contributes to greater instability in the oil−water system.

Furthermore, a comparison of the findings from optical microscopy and IFT analysis reveals key insights that underscore the complementary nature of these techniques in our study. The microscopic images and interfacial tension measurements provide complementary insights into emulsion stability and the role of asphaltenes. Microscopic images reveal how varying oil-to-water ratios impact the formation and stability of emulsions. At higher oil content (75:25), the presence of dispersed water droplets results in a moderate reduction in IFT, indicating partial stabilization by asphaltenes. In contrast, with a balanced ratio of 50:50, the emulsions show more water droplets and greater stability, reflected in a significant reduction in IFT. At this ratio, asphaltenes effectively form barriers at the oil−water interface, leading to lower IFT values and improved stability. When the water content is increased to 25:75, the emulsion exhibits a higher concentration of water droplets. This increased water phase results in a substantial reduction in IFT due to asphaltenes efficiently stabilizing the larger surface area of dispersed water droplets. This ratio demonstrates peak emulsion stability, with asphaltenes playing a key role in reducing IFT and preventing coalescence. The combined analysis of images and IFT values demonstrates that higher water content correlates with increased emulsion stability, as asphaltenes more effectively stabilize the water droplets. The lower IFT values observed at higher water contents confirm the crucial role of asphaltenes in enhancing stability by forming strong interfacial films. By integrating microscopic observations and IFT measurements, the study highlights the critical relationship between asphaltenes, oil−water ratios, and emulsion stability. This interdependence underscores the importance of optimizing these factors to achieve desired stability in water-in-oil emulsions.

### 3.6 Asphaltene Characteristics in Water−Oil Emulsion: FESEM and Elemental Mapping

To examine the changes in dissolved asphaltene characteristics in dead oil after mixing with formation water, the CHN content results of the dissolved asphaltene are presented in Table 7. The results indicate that the nitrogen content in the asphaltene decreases after contact with formation waters. This suggests an interaction between the ions in the injected water and the asphaltene in the oil, potentially leading to alterations in the reservoir fluid properties. Additional tests, including Field Emission Scanning Electron Microscopy (FESEM) and Energy Dispersive X-ray Analysis (EDAX) on the deposits collected from filter paper and the asphaltene separated from live oil, can provide deeper insights into asphaltene precipitation and its impact on emulsion stability.

**Table 7:** CHN Analysis of Dissolved Asphaltene in Dead Oil after Mixing Oil and Formation Water

mixed components	carbon content, C (wt %)	hydrogen content, H (wt %)	nitrogen content, N_2_ (wt %)
blank oil	79.0	7.6	0.7
oil−formation water	77.4	7.9	0.2

**Figure 6:**
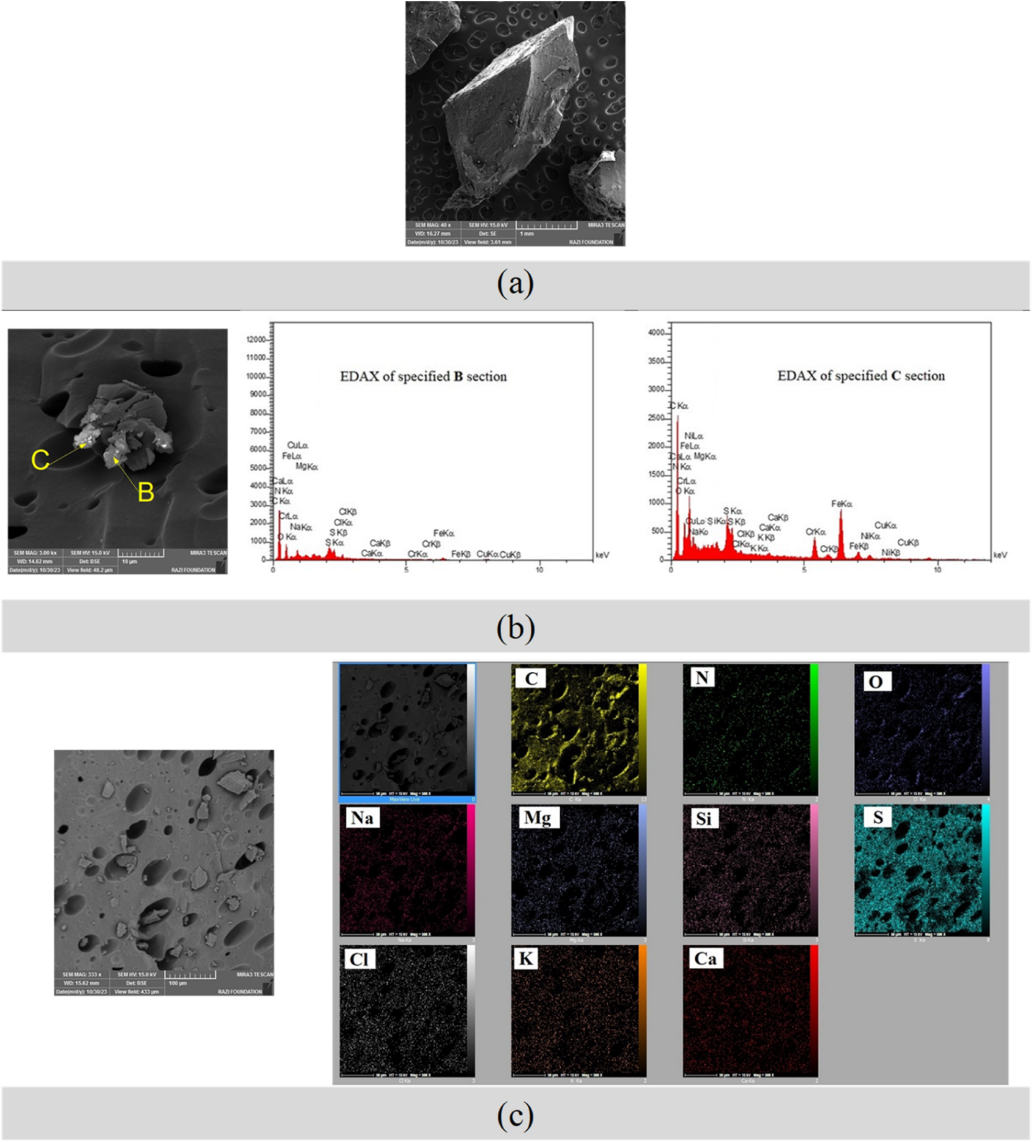
FESEM and EDAX analysis of asphaltene deposit extracted from the blank live oil (free of water emulsion): (a) FESEM micrograph of asphaltene, (b) FESEM micrograph of asphaltene, the two graphs on the right display the EDAX analysis of sections B and C in the asphaltene deposit, (c) EDAX mapping analysis of asphaltene depicted in FESEM (left image).

Figure 6 presents a comprehensive analysis of the asphaltene deposit extracted from blank live oil using FESEM and EDAX techniques. The FESEM micrographs (Figure 6a,b) provide detailed images of the deposit’s morphology, revealing a rough and irregular structure typical of such deposits. These images offer insights into the physical characteristics of the asphaltene, which impact its behavior and stability in oil. EDAX analyses for sections B and C, shown in the accompanying graphs, provide precise elemental compositions, highlighting the presence of elements like carbon, sulfur, and nitrogen. These data suggest the presence of potential impurities or additives and reveal heterogeneities within the asphaltene deposit. Additionally, the EDAX mapping in Figure 6c visualizes the spatial distribution of elements, identifying regions with higher concentrations of specific elements, such as sulfur or metals, which correlate with structural features observed in the micrographs. This detailed mapping is crucial for understanding compositional variations that could influence the properties and processing of the asphaltene.

**Figure 7:**
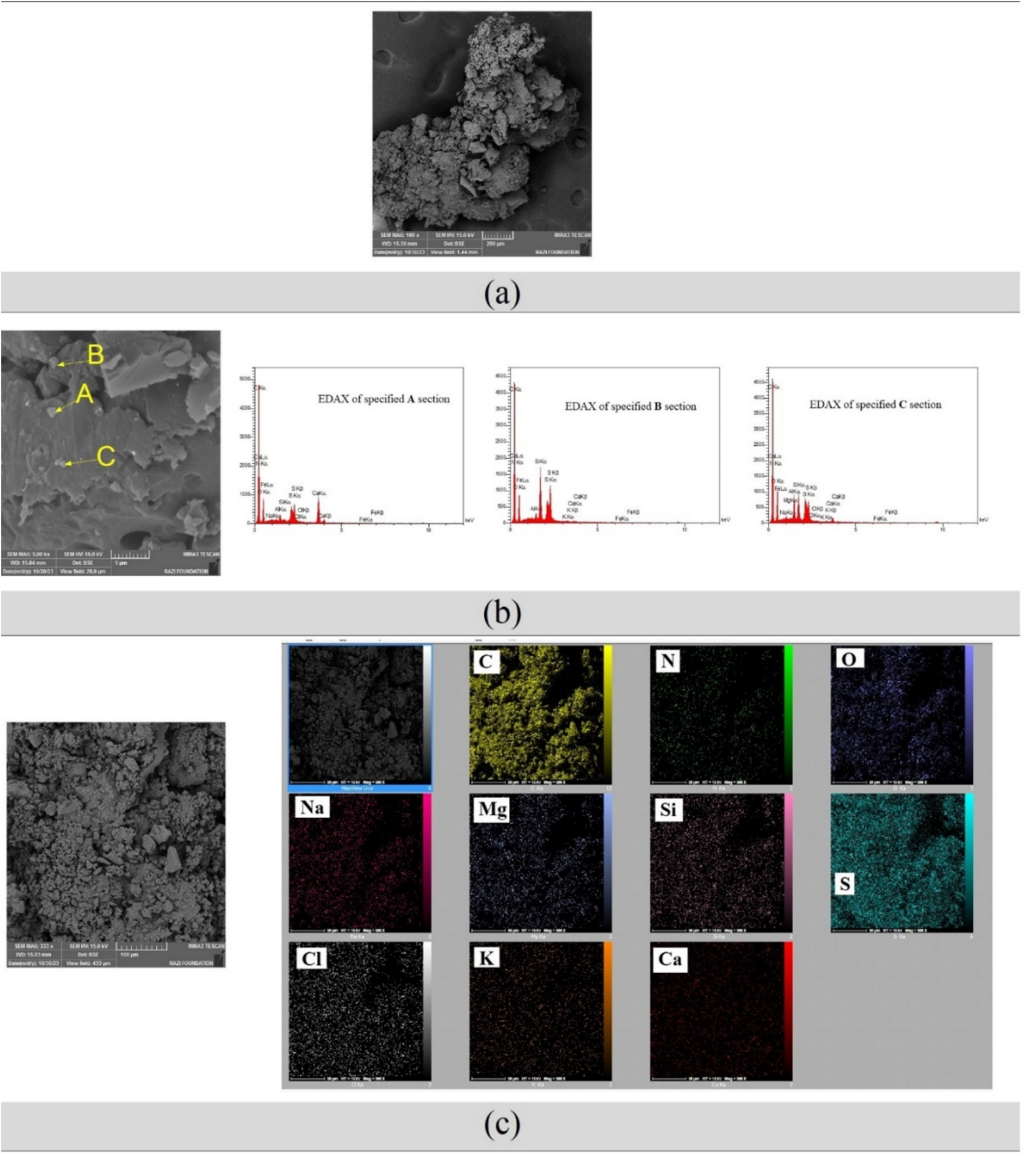
FESEM and EDAX analysis of asphaltene deposit extracted from the live oil including formation water emulsion: (a) FESEM micrograph of asphaltene, (b) FESEM micrograph of asphaltene, the three graphs on the right display the EDAX analysis of sections A, B, and C in the asphaltene deposit, (c) EDAX mapping analysis of asphaltene depicted in FESEM (left image).

Figure 7 presents the FESEM and EDAX analysis of asphaltene deposits extracted from live oil containing formation water emulsion. The FESEM micrographs (Figure 7a,7b) reveal detailed texture and structure of the asphaltene, showing more irregular and porous morphology compared to the asphaltene from blank live oil in Figure 6. The additional perspective in Figure 7b confirms and complements the initial observations, highlighting changes in physical characteristics due to the water emulsion. The EDAX analysis (Figure 7c) for sections A, B, and C details the elemental composition, revealing how water emulsion impacts elemental distribution. The EDAX mapping overlays elemental data onto the FESEM image, highlighting the spatial distribution of elements and identifying areas with higher concentrations of specific elements. When compared to Figure 6, which covers asphaltene from oil without water, Figure 7 underscores the significant influence of emulsified water on altering the morphology and elemental composition of asphaltene deposits.

**Figure 8:**
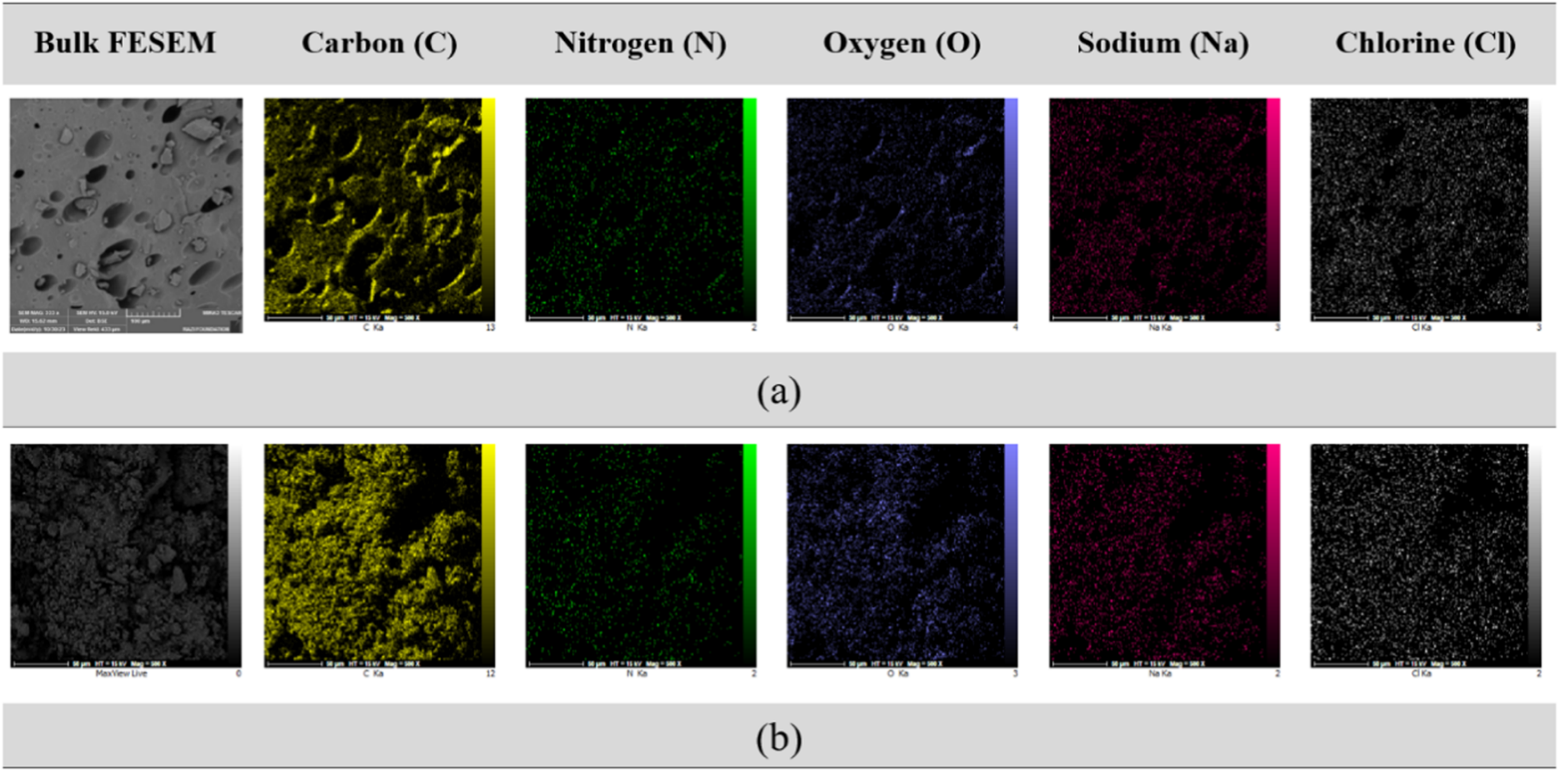
FESEM mapping for asphaltene extracted from the (a) blank live oil, and (b) live oil containing formation water emulsion; for quantitative results refer to Table 8.

For better comparison of the asphaltene structure in blank live oil and in water−live oil emulsion, the FESEM-EDAX mapping are depicted in Figure 8; as well the related quantitative results are given in Table 8. The FESEM mapping and elemental analysis reveal significant differences in asphaltene deposits extracted from blank live oil versus live oil containing formation water emulsion. The asphaltene from live oil with formation water exhibits a more heterogeneous elemental distribution and morphological irregularities compared to the more uniform structure seen in the blank live oil. Quantitative results show increased nitrogen (8.53 wt % vs 6.21 wt %) and oxygen (10.89 wt % vs 7.56 wt %) in the presence of formation water, indicating possible oxidation and interactions with nitrogen compounds. Conversely, elements like sulfur (6.33 wt % vs 8.64 wt %), sodium, magnesium, aluminum, and strontium show decreased concentrations, suggesting dissolution or interaction effects caused by the water emulsion. These findings emphasize the significant impact of formation water on the chemical and physical properties of asphaltene deposits. The physical morphology of asphaltenes influences emulsion stability by affecting their ability to adsorb at the oil−water interface and form stabilizing films. Specifically, rough and porous structures, as observed in the FESEM images (Figures 6−8), provide increased surface area for interaction with water droplets. This allows asphaltenes to form stronger viscoelastic films around water droplets, which act as physical barriers that prevent coalescence. Additionally, the irregular morphology of asphaltenes enhances steric stabilization by creating more effective coverage of the droplets, thereby improving the stability of water-in-oil emulsions. The elemental heterogeneity revealed by EDAX analysis, particularly the presence of elements like sulfur, indicates that these variations may strengthen the stabilizing films, thereby reinforcing the interfacial films and contributing to overall emulsion stability. These insights have been incorporated to address the mechanisms involved.

**Table 8:** Elemental Analysis Results for Asphaltene Deposit Extracted from the Blank Live Oil and Live Oil Containing Formation Water Emulsion

element	blank sample (wt %)	sample with formation water (wt %)
C	75.35	73.20
N	6.21	8.53
O	7.56	10.89
Na	0.34	0.11
Mg	0.25	0.06
Al	0.30	0.10
Si	0.26	0.14
S	8.64	6.33
Cl	0.48	0.27
K	0.10	0.07
Ca	0.08	0.05
Fe	0.09	0.16
Sr	0.34	0.09
**Total**	**100.00**	**100.00**

### 3.7 Practical Implications of the Findings

The insights gained from this study offer several key implications for reservoir management and production operations, particularly in the context of managing asphaltene-related challenges:

- Reservoir Management: A comprehensive understanding of the pressure-dependence of asphaltene precipitation is crucial for the development of effective reservoir pressure maintenance and depletion strategies. The findings indicate that high-pressure conditions suppress asphaltene precipitation, suggesting that maintaining reservoir pressure above critical levels could minimize the risk of asphaltene aggregation and deposition. Additionally, the observed mitigating effect of formation water on asphaltene precipitation highlights the potential for optimizing water flooding strategies. By carefully selecting the composition and volume of injection water, it is possible to enhance oil recovery while simultaneously reducing the risks associated with asphaltene-related plugging and damage to production equipment.
- Production Operations: The study highlights the importance of monitoring and controlling pressure levels during oil production to effectively manage asphaltene precipitation. The reduction in asphaltene deposition in the presence of formation water suggests that water−oil emulsions, particularly those containing live oil, could be leveraged to maintain the stability of asphaltenes and prevent their precipitation. This has significant implications for production operations, as managing the stability of these emulsions can contribute to smoother and more efficient oil production processes. By maintaining optimal pressure conditions and promoting stable emulsions, operators can potentially minimize disruptions and operational risks caused by asphaltene deposition, thereby ensuring sustained production efficiency.

The results of the HPHT filtration experiments clearly demonstrate that asphaltene precipitation is highly sensitive to changes in pressure and the presence of formation water. Elevated pressures were shown to inhibit asphaltene precipitation, while the presence of formation water consistently reduced the quantity of precipitated asphaltene across all tested pressure levels. These findings are pivotal for designing reservoir management and implementation of EOR strategies that align with the specific pressure and fluid characteristics of a given reservoir. By tailoring injection and pressure maintenance protocols to the unique conditions of each reservoir, operators can optimize oil recovery and minimize the risk of asphaltene-related complications.

## 4 Conclusions and Future Directions

This study investigated the stability of water-in-oil emulsions influenced by asphaltene colloids under both atmospheric and HPHT conditions. The results demonstrated that the presence of asphaltenes significantly stabilizes emulsions by forming viscoelastic films at the oil−water interface, which prevents droplet coalescence. Under atmospheric conditions, emulsions were found to be stable across different oil−water ratios, showing resilience over extended periods and highlighting the role of mechanical agitation in maintaining stability. In HPHT scenarios, emulsion stability was observed to be highly dependent on the oil-to-water ratio and pressure conditions. The 75:25 oil-to-water ratio exhibited initial stability that diminished after 24 h, suggesting that additional stabilizing measures may be necessary for long-term effectiveness under reservoir-like conditions. In contrast, higher water content ratios (50:50 and 25:75) consistently produced unstable emulsions, underscoring the challenges associated with water-heavy emulsions in heavy oil reservoirs. Microscopy results revealed the impact of varying oil−water ratios on emulsion stability, indicating that emulsions with higher water content exhibited greater stability due to increased asphaltene interaction at the interface. FESEM and EDAX analyses offered comprehensive details on the morphology and elemental makeup of asphaltene deposits, revealing the substantial effect of formation water on asphaltene properties. Formation water consistently minimized asphaltene precipitation across all pressure conditions, indicating its function as a stabilizing factor. While the polar functional groups of asphaltenes are believed to impart charges to the water droplets, contributing to electrostatic repulsion and preventing coalescence, direct quantification of these charges through ζ-potential analysis was not performed in this study. Future research should include ζ-potential measurements to better understand the electrostatic interactions between water droplets and their contribution to emulsion stability.

The study’s findings provide critical insights into optimizing EOR strategies by adjusting operational parameters such as water flooding rates and pressure maintenance to minimize asphaltene precipitation. Additionally, understanding the role of formation water as a stabilizing agent offers new opportunities to enhance emulsion management practices, particularly in water flooding operations. These results underscore the importance of customizing EOR techniques to specific reservoir conditions − pressure, temperature, oil and water composition, and water content − to achieve efficient oil displacement and recovery. Future research should focus on the long-term behavior and stability of emulsions across a broader range of reservoir conditions to refine strategies. It should also include simulation studies on the interaction of ions in formation water with asphaltenes to gain insights into precipitation and emulsion stabilization mechanisms. While this manuscript emphasizes experimental data, incorporating simulation approaches could further enhance understanding of asphaltene behavior in the presence of ions. Lastly, future studies are encouraged to investigate the effect of increased asphaltene concentrations on emulsion stability to gain deeper insights into the roles of asphaltenes and resins in stabilizing water-in-oil emulsions across various crude oil types.

## Supporting Information Available

The video illustrates the formation of a water-in-oil emulsion in heavy crude oil. It also captures the stability and distribution of water droplets as emulsions, which appear as bubbles of varying sizes dispersed throughout the bulk oil phase. Additionally, it provides a clear depiction of the water−oil interface, highlighting the interaction and stability of the emulsified water within the continuous oil phase. This visual demonstration offers valuable insights into the emulsion characteristics, contributing to a deeper understanding of the system’s behavior (MP4)
